# Injury and cyclic stretch induce vein graft failure: effective treatment with zinc oxide-loaded electrospun polycaprolactone external stent

**DOI:** 10.1093/rb/rbaf119

**Published:** 2025-11-20

**Authors:** Tengzhi Ma, Wenhao Tian, Feixiang Zhu, Yingxin Qi, Kai Huang

**Affiliations:** Key Laboratory for Biomechanics and Mechanobiology of Ministry of Education, School of Biological Science and Medical Engineering, Beihang University, Beijing 100083, China; Key Laboratory for Biomechanics and Mechanobiology of Ministry of Education, School of Biological Science and Medical Engineering, Beihang University, Beijing 100083, China; Institute of Mechanobiology & Medical Engineering, School of Life Sciences & Biotechnology, Shanghai Jiao Tong University, Minhang, Shanghai 200240, China; Key Laboratory for Biomechanics and Mechanobiology of Ministry of Education, School of Biological Science and Medical Engineering, Beihang University, Beijing 100083, China; Institute of Mechanobiology & Medical Engineering, School of Life Sciences & Biotechnology, Shanghai Jiao Tong University, Minhang, Shanghai 200240, China; Institute of Mechanobiology & Medical Engineering, School of Life Sciences & Biotechnology, Shanghai Jiao Tong University, Minhang, Shanghai 200240, China

**Keywords:** external stents, vein grafts, smooth muscle cells (SMCs), intimal hyperplasia, arterialization

## Abstract

The great saphenous vein (GSV) is widely used in vascular surgery, especially for coronary artery bypass grafting (CABG). However, surgical injury and arterial (high) cyclic stretch induce vascular dysfunction in vein grafts. Here, we found that surgical injury induces vascular dysfunctions. Upon adhering to injured vessels, platelets release platelet-derived microvesicles, which serve as potent and persistent mediators of vascular dysfunction. RNA sequencing analysis revealed that zinc ion deficiency plays a vital role in vascular dysfunction. Of note, platelet membrane cloaked Zn-MOF nanoparticles (ZIF-8) alleviate injury-induced vascular dysfunction. To counteract the vascular dysfunction caused by surgical injury and high cyclic stretch in vein grafts, we developed an electrospun polycaprolactone (PCL) external stent loaded with zinc oxide (ZnO) (PCL–ZnO stent). Electrospun PCL external stents containing varying ZnO concentrations (0 wt%, 1 wt%, 3 wt% or 5 wt% ZnO) were fabricated and implanted around vein grafts. Vascular remodeling was assessed by histology, immunofluorescence and RNA sequencing. Moderate ZnO loading (3 wt%) suppressed neointimal hyperplasia to preserve appropriate venous arterialization as confirmed by hematoxylin and eosin (H&E) staining and increased expression of smooth muscle cell phenotypic markers including α-SMA and Calponin. RNA-seq data verified that Zn^2+^ mediates the regulation of genes involved in proliferation, inflammation and metabolism. Gene set enrichment analysis of RNA-seq data from PCL-3 wt% ZnO-treated vein grafts at 2 weeks revealed significant upregulation of gene sets associated with lipid biosynthesis and cholesterol homeostasis. Pathway enrichment analysis of differential metabolites identified significant perturbations in purine metabolism, amino sugar/nucleotide sugar metabolism, galactose metabolism, and glycerophospholipid metabolism. These results indicated that moderate ZnO incorporation (3 wt%) in external stents effectively modulated local biological responses by suppressing pathological cell proliferation without inducing apoptosis, thereby promoting proper venous arterialization. PCL-3 wt% ZnO stent may be a successful material for clinical use in alleviating intimal hyperplasia and promoting functional arterialization of grafted veins.

## Graphical Abstract

**Figure:**
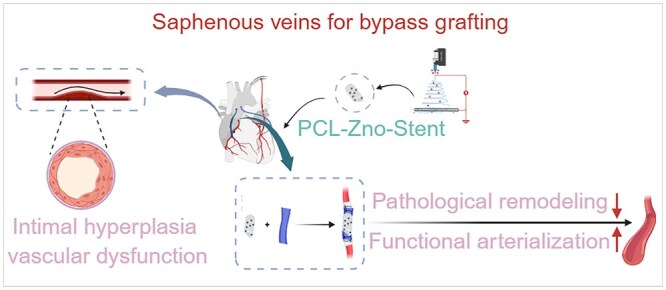

## Introduction

The saphenous vein grafts, particularly the great saphenous vein (GSV), remain a critical therapeutic approach in coronary artery bypass grafting (CABG) for patients with multivessel coronary artery disease [1, 2]. However, upon exposure to arterial hemodynamic forces, vein grafts frequently develop compliance mismatch, intimal hyperplasia and pathological remodeling, resulting in significantly reduced patency over time [3, 4]. This persistent challenge remains a key factor restricting the durability and effectiveness of CABG interventions [5, 6]. Besides, vascular injury also induces intimal hyperplasia and vascular dysfunction after surgery, and the molecular mechanisms need to be further elucidated [7, 8].

Accumulating evidence from others and our laboratory has demonstrated that abnormal mechanical stress serves as a fundamental mediator of endothelial dysfunctions and pathological vascular remodeling [4, 6, 9–17]. In venous grafts, arterial hemodynamics especially high cyclic stretch have been demonstrated to promote neointimal hyperplasia, a key pathological feature of vein graft failure, through activation of the miR-33-BMP3-Smad signaling axis [4]. Furthermore, dysregulated autophagy mediates arterial cyclic stretch-induced neointimal hyperplasia in venous grafts through the p62/Nrf2/SLC7A11 pathway [18]. Tang elucidated that high cyclic stretch also alters vascular smooth muscle cell (VSMC) phenotype via mitochondrial remodeling and metabolic reprogramming. Fan et al. verified that high cyclic stretch-induced cytosolic phospholipase A2 activation impedes fatty acid β-oxidation in vein grafts to promote neointimal hyperplasia [19]. Consequently, enhancing the biomechanical microenvironment of vein grafts to delay or reverse their pathological progression has emerged as a critical research focus in cardiovascular surgery and biomaterials science.

Zinc-based biomaterials surpass first-generation magnesium (rapid corrosion) and second-generation iron (incomplete resorption) stents, emerging as the leading third-generation biodegradable metal for vascular applications [20–24]. Zinc plays essential biological roles as a cofactor in numerous enzymatic and signaling pathways, contributing to angiogenesis, oxidative stress defense, cell migration and immune regulation [24, 25]. Within physiological concentrations, zinc promotes proliferation, migration and adhesion of vascular endothelial cells and smooth muscle cells (SMCs) [26, 27]. When applied in external stent, zinc-based materials not only provide biodegradable structural support but also release zinc in a controlled manner, endowing them with anti-inflammatory, anti-proliferative and endothelial-protective properties [28]. Furthermore, 3D atherosclerotic vascular models have demonstrated that zinc exerts dual regulatory effects in inflammatory environments, offering concentration-dependent protection or damage to endothelial cells [25]. However, excessive zinc concentrations exhibit biphasic toxicity, inducing apoptosis, oxidative damage and inflammatory activation [29, 30]. Zinc-based external stent may not only offer mechanical support but also dynamically modulate local biological behaviors, particularly during the high-risk postoperative window. These features make them promising candidates for next-generation bioresorbable vascular stents.

**Figure 1.**
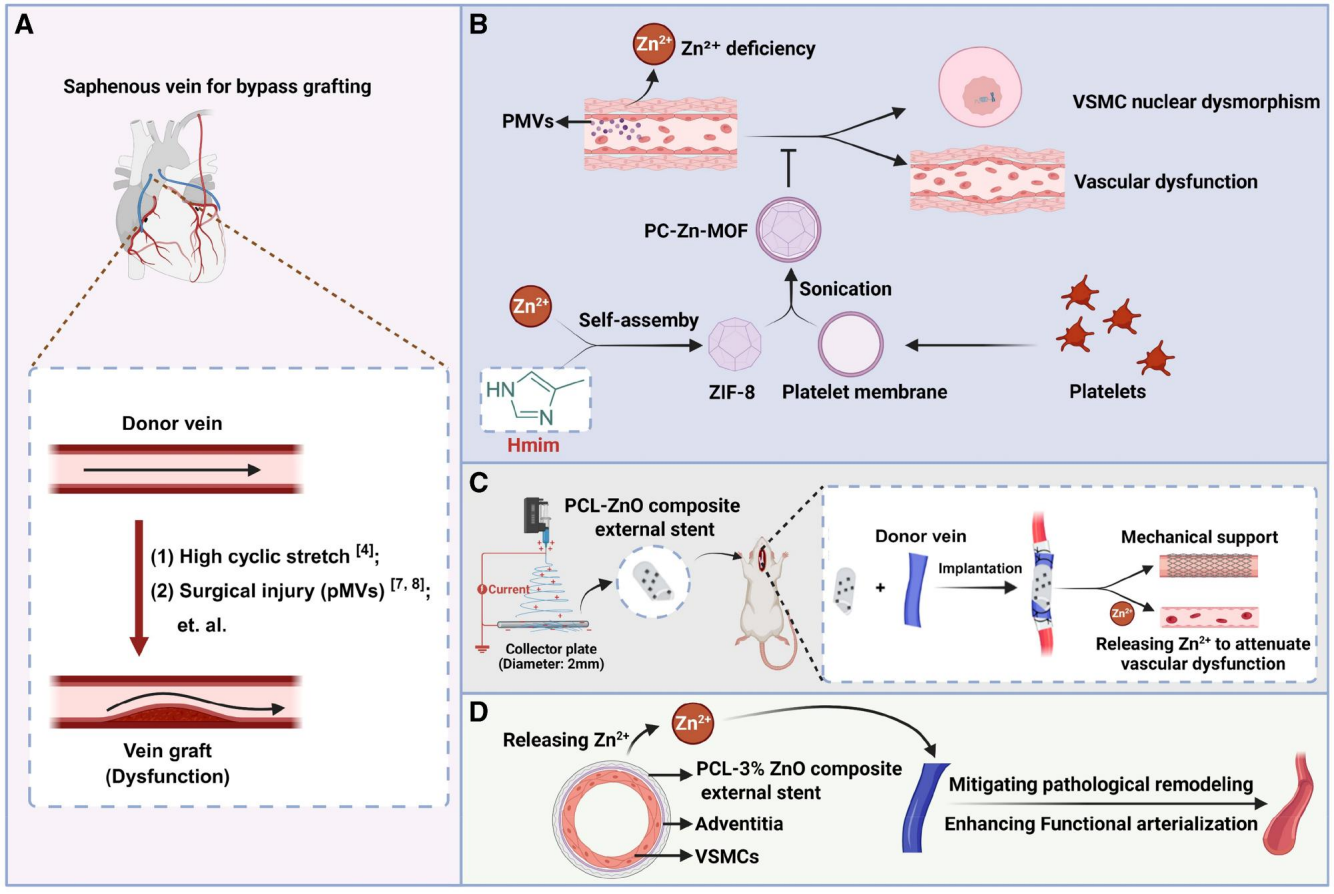
Research framework and methodology flow diagram. (**A**) Surgical injury and high cyclic stretch contribute to vascular dysfunction in vein grafts. (**B**) PMVs induced Zn^2+^ deficiency promotes vascular dysfunction after injury and platelet membrane cloaked Zn-MOF nanoparticles (ZIF-8) alleviates vascular dysfunction post-injury. (**C**) Zinc oxide (ZnO)-loaded electrospun polycaprolactone (PCL) external stents (PCL–ZnO stents) were engineered to offer mechanical support while simultaneously releasing zinc ions. (**D**) PCL-3 wt% ZnO stents effectively mitigate pathological remodeling and enhance functional arterialization of vein grafts.

By targeting both surgical injury and hemodynamic-induced arterial cyclic stretch (Figure 1A), we developed combinatorial interventions to counteract vascular dysfunction in vein grafts, offering a clinically viable approach to enhance patency. We demonstrated that platelet-derived microvesicles (PMVs) adhere to injured arterial walls, inducing Zn^2+^ deficiency in VSMCs, which triggers nuclear dysmorphism and subsequent vascular dysfunction (Figure 1B). Supplementation with platelet membrane cloaked Zn-MOF nanoparticles (ZIF-8) effectively restores vascular function post-injury by addressing Zn^2+^ deficiency in VSMCs (Figure 1B). Accordingly, we engineered ZnO-loaded electrospun polycaprolactone (PCL) stents (PCL–ZnO stents) that provide mechanical reinforcement while releasing Zn^2+^ to mitigate vein graft dysfunction (Figure 1C). Unlike PCL-1% ZnO and PCL-5% ZnO stents, the PCL-3 wt% ZnO stent demonstrates superior efficacy in mitigating pathological remodeling and promoting functional arterialization of vein grafts (Figure 1D). Therefore, PCL–ZnO stent represents a novel functional biomaterial with therapeutic potential for vein graft dysfunction.

## Materials and methods

### Human injured vessels

Informed consent was obtained from all participants prior to the study. The experimental protocols involving human femoral artery and posterior tibial artery samples were reviewed and approved by the Ethics Committee of Shanghai Ninth People’s Hospital, Shanghai Jiao Tong University School of Medicine (approval ID: SH9H-2021-TK32-1). All procedures adhered to the ethical principles of the Declaration of Helsinki.

### Animals

The animal care and experimental procedures were conducted in accordance with the Animal Management Rules of China (55, 2001, Ministry of Health, China), as well as the guidelines outlined in Directive 2010/63/EU of the European Parliament for the protection of animals used for scientific purposes. The study was approved by the Animal Research Committee of Shanghai Jiao Tong University.

### Rat carotid artery intimal injury model

Male Sprague–Dawley rats, weighing 320 ± 20 g, were anesthetized with 2% isoflurane administered at a 1.8 L/min oxygen flow using an isoflurane vaporizer (MATRX VIP 3000, USA). The left carotid arteries were exposed, and vascular intimal injury was induced using a percutaneous transluminal angioplasty balloon dilatation catheter (2 F, 0.67 mm; Edwards Lifesciences, USA). Arteries were harvested after 1, 3, 7 and 14 days, with the intact right carotid artery serving as the self-control. Rats were euthanized by CO_2_/O_2_ inhalation in all experiments.

### Immunofluorescence staining

Paraffin sections were deparaffinized and permeabilized with 0.3% Triton X-100 for 30 min, followed by three washes with PBS. Antigen retrieval was carried out in 10 mM sodium citrate buffer (pH 6.0) by microwave heating at boiling temperature for 15 min. After cooling to room temperature (RT), the sections were washed with PBS and incubated with 10% goat serum for 30 min at RT to block nonspecific binding. For cellular staining, VSMCs were fixed with paraformaldehyde for 30 min, permeabilized with 0.3% Triton X-100 for 30 min, and then stained as described above.

The sections or cells were then incubated overnight at 4°C with the following primary antibodies: Ki-67 (1:1000; Wuhan Sevier Biotechnology, China), CD41 (1:100; Abcam, Cambridge, UK), α-SMA (1:1000; Proteintech, China), Lamin A (1:200; Santa Cruz Biotechnology, Dallas, TX, USA), H3K9me3 (1:2000; Cell Signaling Technology, USA), H3K9ac (1:2000; Cell Signaling Technology, USA) or Calponin (1:1000; Proteintech, China). The next day, sections or cells were incubated with an appropriate secondary antibody (1:1000; Abcam, England) for 2 h at RT. Nuclei were counterstained with DAPI for 15 min at RT. Fluorescence images were captured using a confocal laser scanning microscope (Olympus Confocal-FV1000, Japan).

### Apoptosis assay

Dewaxed and rehydrated sections were digested with Proteinase K (37°C, 20 min), permeabilized (0.3% Triton X-100, 30 min) and equilibrated (10 min, RT). TUNEL assays were performed using a commercial kit (Wuhan Sevier Biotechnology), with TdT/dUTP/buffer (1:5:50) incubated at 37°C (60 min). After PBS washes and DAPI counterstaining (15 min, RT), TUNEL^+^ cells were quantified by ImageJ.

### Western blotting

Frozen arterial or venous graft tissue (5 mg) was homogenized in 50 μl RIPA lysis buffer. SMCs were washed with cold PBS and lysed in RIPA buffer at 4°C for 5 min. After centrifugation (10 000 × g, 10 min, 4°C), protein concentrations were determined using a BCA assay.

Proteins were separated by 10% SDS-PAGE and transferred to PVDF membranes (Millipore). Membranes were probed with primary antibodies against α-SMA (1:2000; Proteintech), Calponin (1:2000; Proteintech), SM22 (1:2000; Proteintech) and GAPDH (1:2000; Santa Cruz Biotechnology), followed by incubation with AP- or HRP-conjugated secondary antibodies (Jackson ImmunoResearch). AP signals were detected with NBT/BCIP (Bio Basic), while HRP signals were visualized using ECL (Bio-Rad).

### RNA sequencing

Total RNA was isolated from rat arteries or vein grafts using TRIzol reagent (Invitrogen) following the manufacturer’s protocol. RNA quality and concentration were assessed using an Agilent Bioanalyzer 2200. High-quality RNA samples were used to construct cDNA libraries with the NEBNext^®^ Ultra™ Directional RNA Library Prep Kit (Illumina). Libraries were sequenced on an Illumina HiSeq X Ten platform, generating 150 bp paired-end reads. Reads were aligned to the human genome (GRCh38) using HISAT2, and gene expression levels (FPKM) were quantified with Cufflinks. Read counts were obtained via HTSeq-count, and differential expression analysis was performed using DESeq (R package, 2012), with *P *< 0.05 set as the significance threshold.

### Gene set enrichment analysis

Functional enrichment of transcriptomic data was evaluated via gene set enrichment analysis (GSEA) implemented in GSEA v4.1.0 (Broad Institute). Enrichment significance was determined using an FDR threshold of <0.05.

### Cell culture

Primary SMCs were isolated from the thoracic aortas of male Sprague–Dawley rats [12, 17, 31]. The aortic medial layer was dissected aseptically, minced into fragments, and cultured in DMEM (Gibco) supplemented with 10% FBS (Gibco), 100 U/mL penicillin, and 100 μg/mL streptomycin (37°C, 5% CO_2_). Cells were passaged every 3–4 days upon reaching confluence, and passages 4–7 were used for experiments. Each experiment employed freshly isolated SMCs from different rats. Venous SMCs were isolated and cultured under identical conditions, except the medium contained 20% FBS [4].

### Nuclear morphology measurements

Nuclear morphology parameters—including Compactness, Eccentricity, Formfactor, Solidity and Zernike shape features—were quantified using CellProfiler (v4.2.5) [32]. Measurements were performed via the Measure Object Size Shape and Measure Texture modules following nuclear segmentation.

### Transmission electron microscope analysis

SMCs and vessels were initially fixed with 3% glutaraldehyde, followed by post-fixation using 2% osmium tetroxide. After embedding in resin, semi-thin epoxy sections were observed under a light microscope (UC6-FC6, Leica, Germany) to precisely localize regions of interest for ultrathin sectioning. Sections with a thickness of approximately 70 nm were obtained and mounted onto formvar-coated copper grids, then left to dry overnight. Subsequently, the sections were stained with uranyl acetate for 30 min, rinsed with PBS and counterstained with lead citrate for 7 min. Transmission electron microscope (TEM) images were acquired using a Talos L120C G2 TEM (Thermo Fisher Scientific, Waltham, MA, USA).

### Ingenuity pathway analysis

Ingenuity pathway analysis (IPA, Qiagen; https://www.qiagenbioinformatics.com/products/ingenuity-pathway-analysis) was employed to identify potential biological processes and functional annotations. This software leverages a curated knowledge base derived from peer-reviewed literature, encompassing information on genes, proteins, molecular interactions, drugs, chemicals, signaling pathways and biological functions. By integrating this comprehensive dataset, IPA enables in-depth exploration of complex biological networks and chemical interactions, facilitating mechanistic insights through evidence-based or predictive modeling approaches.

### Bulk RNA sequencing data analysis

R package “GEOquery” (version 2.70.0) was used to get intimal injury datasets GSE164050 and vein graft datasets GSE241205 from the GEO database (https://www.ncbi.nlm.nih.gov/geo/). GSE241205 included gene expression data for three occluded vein grafts and three intraoperative spare GSVs from three patients undergoing clinical re-CABG. GSE164050 included gene expression data for four arteries with intimal injury and four control arteries from the left carotid arteries of Sprague–Dawley rats. We utilized the R package “DESeq2” (version 1.46.0) to do the differential gene analysis, and the threshold of |log_2_(Fold change) | > 1 and adjusted *P *< 0.05 was chosen as the threshold to identify differentially expressed genes (DEGs) in these datasets. Based on these DEGs, we conducted GO analysis using the “GOplot” package (version 1.0.2).

### High-zinc diet

All mice were maintained on a standard diet containing 30 ppm zinc ions (Dyets Inc., Bethlehem, PA, USA). Following intimal injury, the animals were switched to a high-zinc diet (150 ppm zinc ions; Dyets Inc., Bethlehem, PA, USA). Control group mice continued to receive the standard diet throughout the study.

### Platelet membrane cloaked Zn-MOF nanoparticles

Zinc nitrate hexahydrate (Sigma-Aldrich; final concentration: 1 mg/mL) and 2-methylimidazole (Sigma-Aldrich; final concentration: 20 mg/mL) were vigorously mixed for 30 s and then incubated undisturbed for 3 h to synthesize Zn-MOF nanoparticles. The morphology of the nanoparticles was characterized using TEM.

Platelet membranes were isolated by repeated freeze–thaw cycles followed by low-speed centrifugation (1000 × g) to remove intracellular contents. The purified platelet membranes were then co-incubated with Zn-MOF nanoparticles and subjected to ultrasonication (42 kHz, 2 min) to produce platelet membrane-coated Zn-MOF nanoparticles (ZIF-8).

### Fabrication of ZnO-loaded electrospun PCL external stent (PCL–ZnO stent)

PCL pellets (Mw = 70 000–90 000) and zinc oxide (ZnO, 99.9%) were purchased from Sigma-Aldrich (Shanghai, China). ZnO was first dissolved in a methanol–chloroform mixture (1:5, v/v). Subsequently, PCL pellets were added to the solution and stirred continuously at RT for up to 12 h to obtain a homogeneous electrospinning solution. The final concentration of PCL in the solution was 15% w/v, while the concentrations of ZnO were adjusted to 0%, 1%, 3% and 5% w/v, respectively, and denoted as PCL-0 wt% ZnO, PCL-1 wt % ZnO, PCL-3 wt % ZnO, and PCL-5 wt % ZnO. Electrospun stents were fabricated using a stainless-steel rotating rod collector (2 mm diameter, 150 rpm). The polymer solution was loaded into a 10-ml glass syringe connected to a syringe pump (Cole-Parmer, USA). Electrospinning was carried out under the following parameters: flow rate of 2 mL/h, applied voltage of 13 kV, and needle gauge of 21 G. The resulting stents were vacuum-dried at RT for up to 3 days to remove residual solvents. Prior to further experimental use, the stents were sterilized with ethylene oxide gas.

### PCL–ZnO stent morphological characterization

The morphology of pure PCL stents and ZnO-loaded stents was examined using scanning electron microscopy (SEM; Phenom Pro, Phenom-World BV) operated at an accelerating voltage of 15 kV. Prior to imaging, samples were sputter-coated with gold to enhance conductivity. SEM images were analyzed using Image-Pro Plus software (IPP; Media Cybernetics, Rockville, MD, USA) to quantify the luminal diameter, wall thickness, fiber diameter and pore size of the grafts. The microstructure and elemental composition of the PCL–ZnO stent were examined using a Zeiss ULTRA PLUS SEM (Oberkochen, Germany), which was equipped with an energy dispersive X-ray spectroscopy (EDS) detector and operated at an accelerating voltage of 15 kV.

### Mechanical characterization

Radial mechanical performance was assessed using a tensile testing machine with a 100 N load cell (Instron 3345; Norwood, MA). Graft segments approximately 0.3 cm in length were mounted between two steel rings secured by the machine clamps and stretched at a rate of 10 mm/min until failure. Tensile strength and ultimate elongation at break were recorded, while Young’s modulus was calculated from the initial linear region (≤5% strain) of the stress–strain curve.

### Wide angle X-ray diffraction analysis

The conformation of PCL, and phase structure of ZnO nanoparticles and PCL/ZnO nanocomposites with diverse content of ZnO (0, 1, 3 and 5 wt%) were evaluated using an X-ray diffractometer (XRD; PANalytical, The Netherlands) at 40 kV and 40 mA. All patterns were scanned using CuKα radiation from 10 to 80° in 2θ.

### Infrared spectroscopy

In order to detect changes at the functional groups of our stents after degradation, Fourier Transform Infrared (FTIR) spectroscopy was accomplished. An FTIR device model Nicolet 5700 (Thermo Nicolet, USA) equipped with a DTGS detector, and an air purge system (Parker-Balston, USA) was used. Samples were cut and hold on an air window to measure infrared transmission between 400 and 4000 cm^−1^. Spectra were obtained with 2 cm^−1^ resolution and after 32 iterations.

### Release kinetics of zinc from stents

The release kinetics of zinc from the prepared stents were studied. Stents were cut into samples 0.5 cm in length. Each stent was immersed in 10 ml of PBS in a sealed container and incubated at 37°C. At predetermined time intervals, 0.2-mL aliquots were withdrawn and analyzed for the concentration of zinc using flame atomic absorption spectrophotometry (Shimadzu, AA-7000; Japan).

### *In vitro* biocompatibility

For cell viability assays, fibrous scaffold sections (*n* = 3) of 1 cm^2^ were transferred to a 24-well plate and UV sterilized for 2 h (1 h per side). The stents were washed with D-PBS and seeded with 1 × 10^4^ SMCs in 1 ml complete media. Cell viability on the stents was assessed using the CCK-8 assay after 1, 3 and 5 days of incubation. At each time point, the media in the wells was replaced with 500-μl fresh plain media and 50-μL CCK-8 solution. The plates were incubated for 2 h at 37°C and 5% CO_2_. Absorbance was measured at 450 nm using a microplate reader (Eon, Biotek, USA).

### Vein graft rat model

Vein grafting was performed in normal adult rats using the “cuff” technique [4, 33]. Male Sprague–Dawley rats (8–10 weeks, 250–300 g) were anesthetized with 2% isoflurane (1.8 L/min O_2_) and maintained on a heating pad. Under sterile conditions, the external jugular vein was isolated via a 2–3 cm neck incision, ligated (7-0 silk sutures), and excised into cold heparinized saline (50 U/mL). The left common carotid artery was transected, and polyimide cuffs (1 ± 0.2 mm ID) were fitted to its ends. The vein graft was sleeved over the cuffed artery and secured with 8-0 silk sutures. Blood flow restoration was confirmed immediately. Grafts were harvested at 1-, 2- and 4-week post-operation for analysis. All procedures followed institutional ethical guidelines (Shanghai Jiao Tong University Animal Ethics Committee approval).

### H&E staining

The harvested vessels were fixed in 4% paraformaldehyde for 24 h, rinsed with PBS and subjected to graded dehydration before paraffin embedding. Serial sections (5 μm thickness) were prepared, deparaffinized and rehydrated for standard H&E staining. Histological evaluation was performed using light microscopy, with representative images captured for structural analysis.

### Metabolite extraction and analysis

Two-week vein grafts (weighed 0.1 g) were ground with liquid nitrogen, and extracted using 120 μL of pre-cooled 50% methanol (MeOH:H_2_O = 1:1). A 20-μL aliquot was vortexed for 1  min, incubated at RT for 10 min and stored overnight at –20°C. Samples were then centrifuged (4000 × g, 20  min), and supernatants were chromatographically separated using an ACQUITY UPLC T3 column (100 mm  ×  2.1 mm, 1.8  μm; Waters, UK) at 35°C, with solvent A (water, 0.1% formic acid) and solvent B (acetonitrile, 0.1% formic acid). Mass spectrometry was performed on a triple TOF 5600 system (SCIEX, UK) in IDA mode (m/z 60–1200 Da).

Data were converted to mzXML and processed with XCMS for peak detection, alignment and annotation. MetaX was used for metabolite identification (matching m/z within 10 ppm to KEGG and HMDB). PCA and PLS-DA assessed data quality and outliers. *P* values <0.05 were set as the threshold for significantly differential expression.

### Statistical analysis

Data are presented as mean ± standard deviation. Statistical analyses were performed using GraphPad Prism software (version 8.1; GraphPad, San Diego, CA). For comparisons between two groups, Student’s *t*-test was used. For comparisons among multiple groups, data normality was first assessed using the Shapiro–Wilk test, followed by one-way analysis of variance with Bonferroni’s *post hoc* multiple comparisons. The *P* values <0.05 were considered statistically significant. In all figures, statistical significance is indicated as follows: *P *< 0.05 (*), *P *< 0.01 (**) and *P *< 0.001 (***).

## Results

### Surgical injury induces vascular dysfunctions

**Figure 2.**
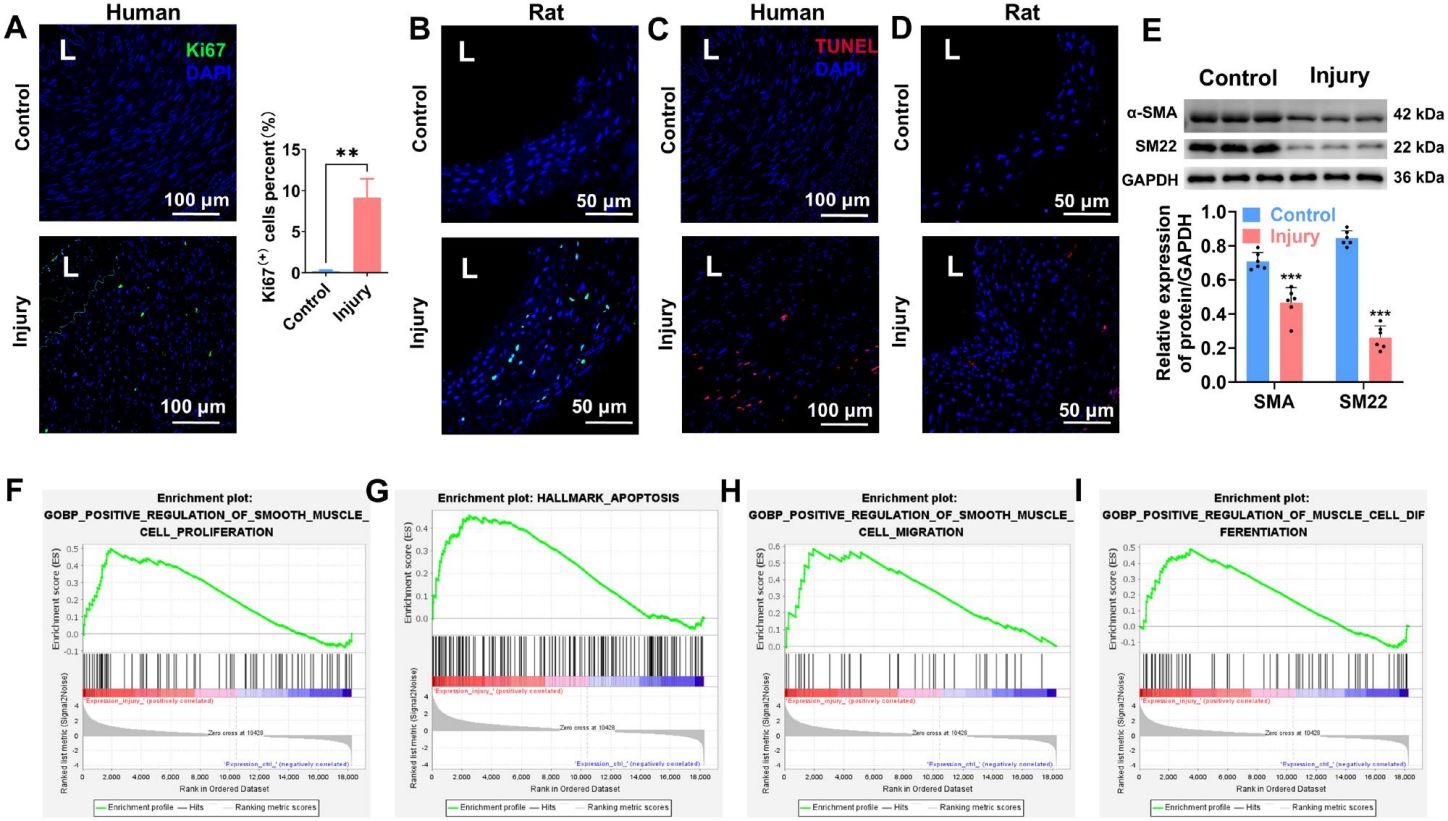
Surgical injury induces vascular dysfunctions. (**A**) IF staining for Ki67 (green) and DAPI (blue) in human intact and injured samples, indicating VSMC abnormal proliferation after injury. Quantification of Ki67^+^ cells is presented at right. (**B**) IF staining for Ki67 in rat control and injury samples, showing excessive proliferation of VSMCs in rat post-injury. (**C**) TUNEL staining (red) in human control and injury samples, with DAPI staining the nuclei (blue). (**D**) TUNEL staining in rat control and injury samples, confirming increased apoptosis rate in the injured group. (**E**) Western blot analysis of α-SMA and SM22 expression in rat samples, with quantitative analysis showing decreased expression of these markers in the injury group compared to controls. GSEA of RNA-seq data enriched the pathways related to aberrant proliferation (**F**), apoptosis (**G**), migration (**H**) and differentiation (**I**). These results reflected the critical biological processes underlying VSMC dysfunctions. The data are shown as mean ± SD. Comparisons between two groups were performed using paired Student’s *t*-test. Significance was indicated as **P < 0.01, ***P < 0.001.

Injured vessels were harvested from both humans and rats. IF staining for Ki67 (green) revealed that injury-induced cell proliferation in both human (Figure 2A) and rat (Figure 2B) samples. TUNEL staining (red) was used to detect apoptotic cells, and the results showed a higher number of TUNEL-positive cells in the injured vessels both in human (Figure 2C) and rat (Figure 2D) samples compared to controls, indicating an increase in cell apoptosis rate following injury. To assess the impact of injury on SMC differentiation, western blot analysis was performed to measure the expression of α-SMA and SM22. The results showed a significant reduction of both α-SMA and SM22 in the injury groups compared to controls (Figure 2E). These results indicate that injury impairs SMC differentiation.

GSEA was further used to explore the impact of injury on SMC functions. GSEA of RNA-seq data from injured vessels enriched the pathways related to cell proliferation (Figure 2F), apoptosis (Figure 2G), migration (Figure 2H) and differentiation (Figure 2I). These results reflected the critical biological processes underlying VSMC dysfunctions post-injury.

### Platelets induce VSMC nuclear dysmorphism after injury

**Figure 3.**
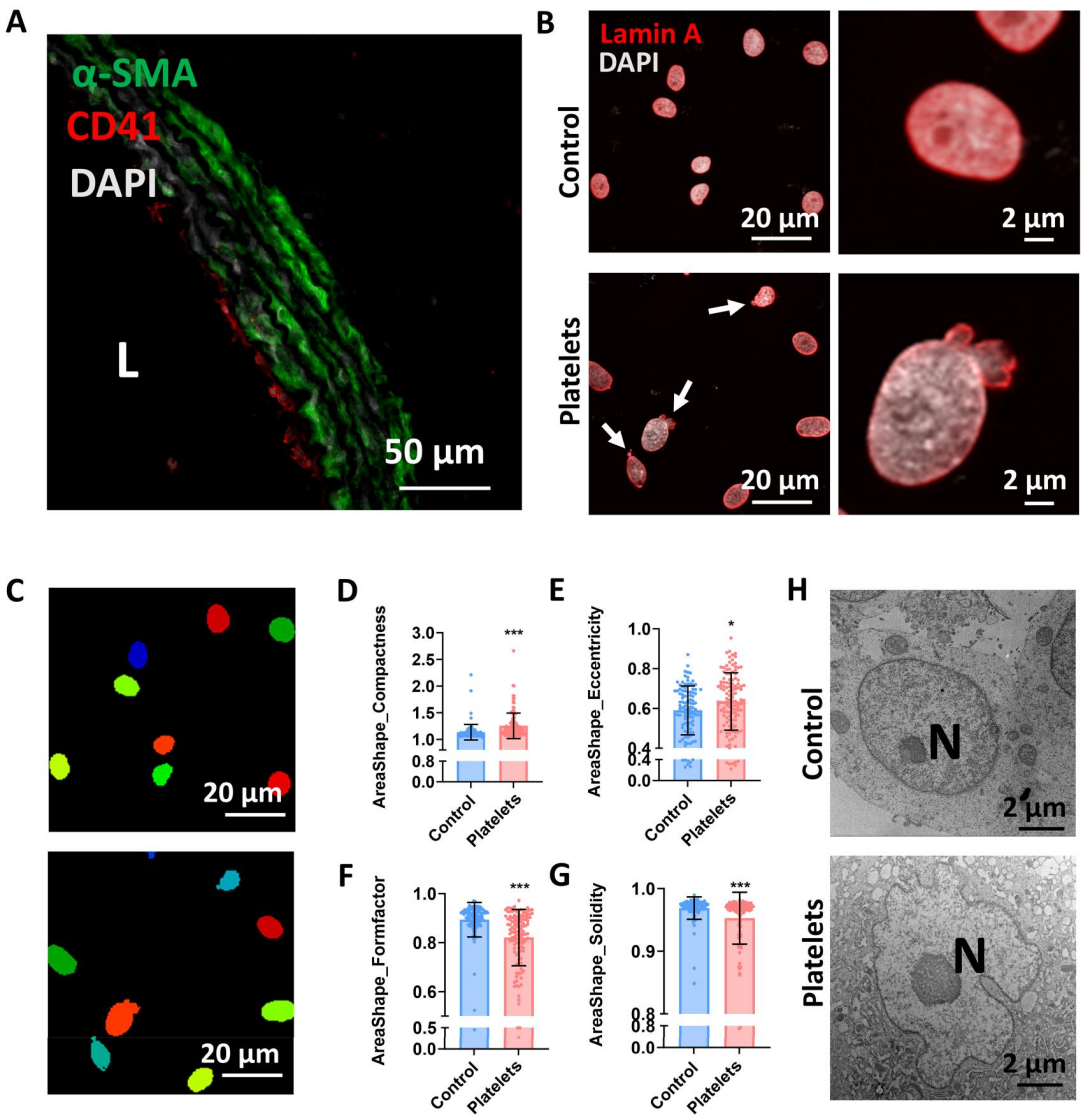
Platelets induce VSMC nuclear dysmorphism. (**A**) Representative immunofluorescence image showed that platelets adhered to injured arteries *in vivo*. Vascular smooth muscle cell marker α-SMA (green) and the platelet marker CD41 (red) were co-stained in injured carotid arteries, and nuclei were counterstained with DAPI. (**B**) Representative immunofluorescence staining of nuclei co-stained with DAPI (white) and Lamin A (red) in VSMCs after platelets treatment. Arrows indicate the deformed nuclei. (**C**) Representative images of nuclear morphology analyzed by CellProfiler, indicating nuclear dysmorphism in VSMCs co-cultured with platelets. Compactness (**D**), Eccentricity (**E**), Formfactor (**F**), and Solidity (**G**) quantified the nuclear morphology of VSMCs co-cultured with platelets (the number of nuclei >100). (**H**) Representative images of the nuclei captured by TEM, and nuclear dysmorphism was detected in VSMCs co-cultured with platelets. The data are shown as means ± SD, *P < 0.05, ***P < 0.001 (Student’s *t*-test). *n* = 3. L, lumen; N, nucleus.

Our findings establish that platelet activation and PMVs release act as persistent triggers of vascular dysfunction, both in acute intimal injury and hypertensive conditions [14, 34]. Given that platelets adhered to the injured artery *in vivo* within 1 h (Figure 3A), whether platelets contribute to the formation of VSMC nuclear dysmorphism was investigated. As shown in Figure 3B, nuclear dysmorphism was captured in VSMCs after being co-cultured with platelets. CellProfiler v4.2.5 [32] software was used to measure nuclear morphology (Figure 3C) after platelet treatment. The parameters including Compactness, Eccentricity, Formfactor and Solidity were used to assess nuclear morphology. Compactness (Figure 3D) and Eccentricity (Figure 3E) were robustly increased, while Formfactor (Figure 3F) and Solidity (Figure 3G) were strongly decreased after platelet treatment. Besides, TEM images further validated nuclear dysmorphism (Figure 3H) after platelet treatment.

Therefore, platelets rapidly adhere to injured vessels and induce nuclear dysmorphism in SMCs.

### PMVs induce SMC nuclear dysmorphism and dysfunctions

**Figure 4.**
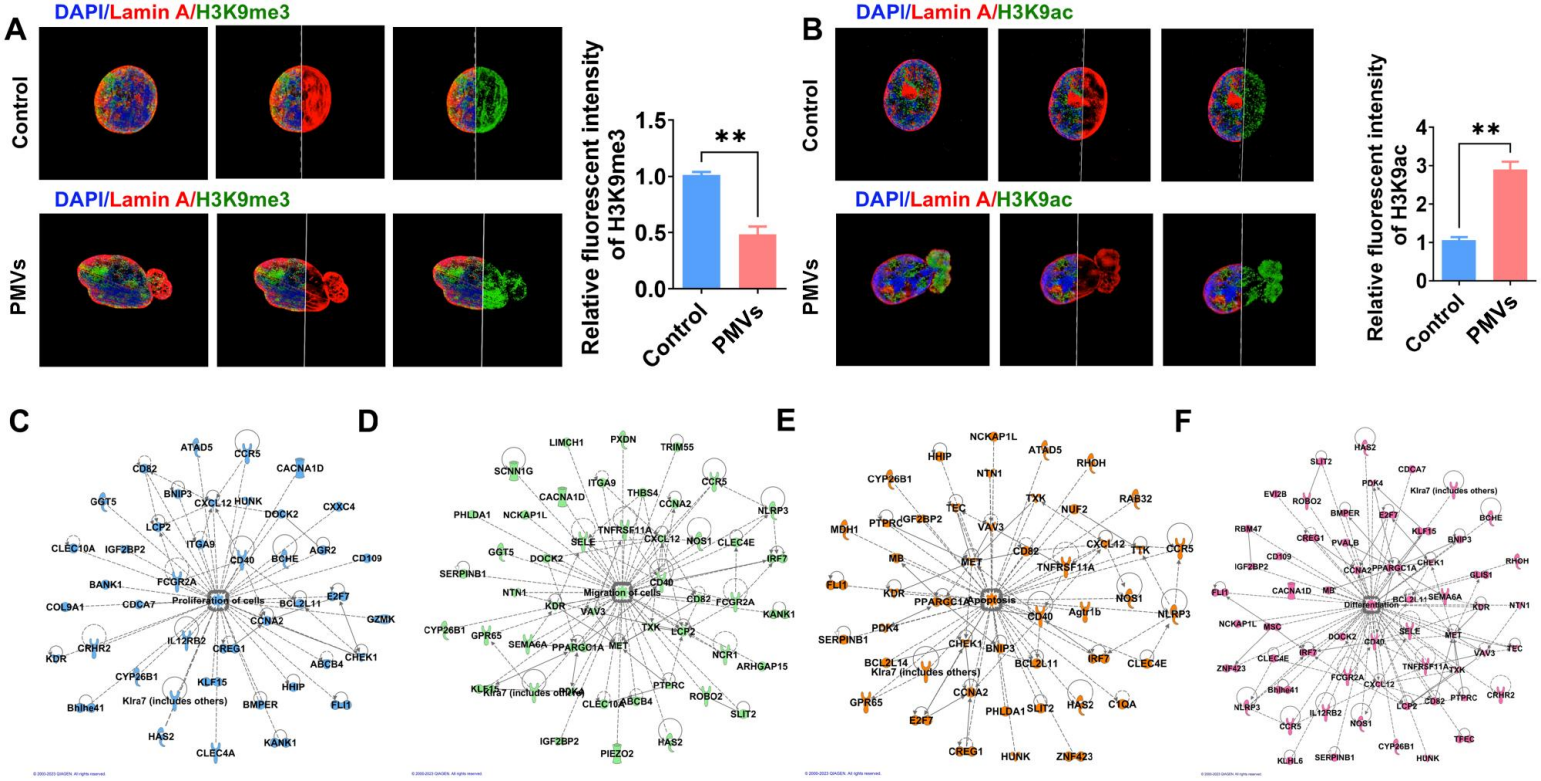
PMVs induce VSMC nuclear dysmorphism and vascular dysfunctions. (**A**) Structured illumination microscopy (SIM) images observed Lamin A (red), H3K9me3 (green) and DAPI (blue) fluorescence intensity in VSMC nuclei after PMVs treatment which showed a notable decline of H3K9me3 expression in the blebs of nuclei. Quantification of relative fluorescence intensity is shown at right. (**B**) SIM images showed Lamin A (red) and H3K9ac (green) immunofluorescence intensity after PMVs treatment, H3K9ac expression was increased in the blebs of nuclei. Quantification of relative fluorescence intensity is shown at right. (**C**–**F**) Ingenuity pathway analysis (IPA; Qiagen, Venlo, Netherlands) showed that genes within LADs participate in proliferation (**C**), migration (**D**), apoptosis (**E**), and differentiation (**F**). Significance was indicated as **P < 0.01.

PMVs were isolated as we previously described [14, 15, 31]. IF staining showed that PMV treatment induces nuclear dysmorphism accompanied by reduced H3K9me3 expression (Figure 4A). 3D reconstruction observation captured a conspicuous decline of H3K9me3 expression in the blebs of the nucleus after PMV treatment. However, H3K9ac was highly expressed in the blebs (Figure 4B) after PMV treatment. Reduced H3K9me3 and elevated H3K9ac levels suggest aberrant genes activation in SMCs following PMVs treatment. IPA further verified that the abnormal activated genes were linked to hyperactive cell functions including proliferation (Figure 4C), migration (Figure 4D), apoptosis (Figure 4E) and differentiation (Figure 4F). Therefore, PMVs induce SMC nuclear dysmorphism and cell dysfunctions.

### Effects of zinc on SMC functions *in vitro* and *in vivo*

**Figure 5.**
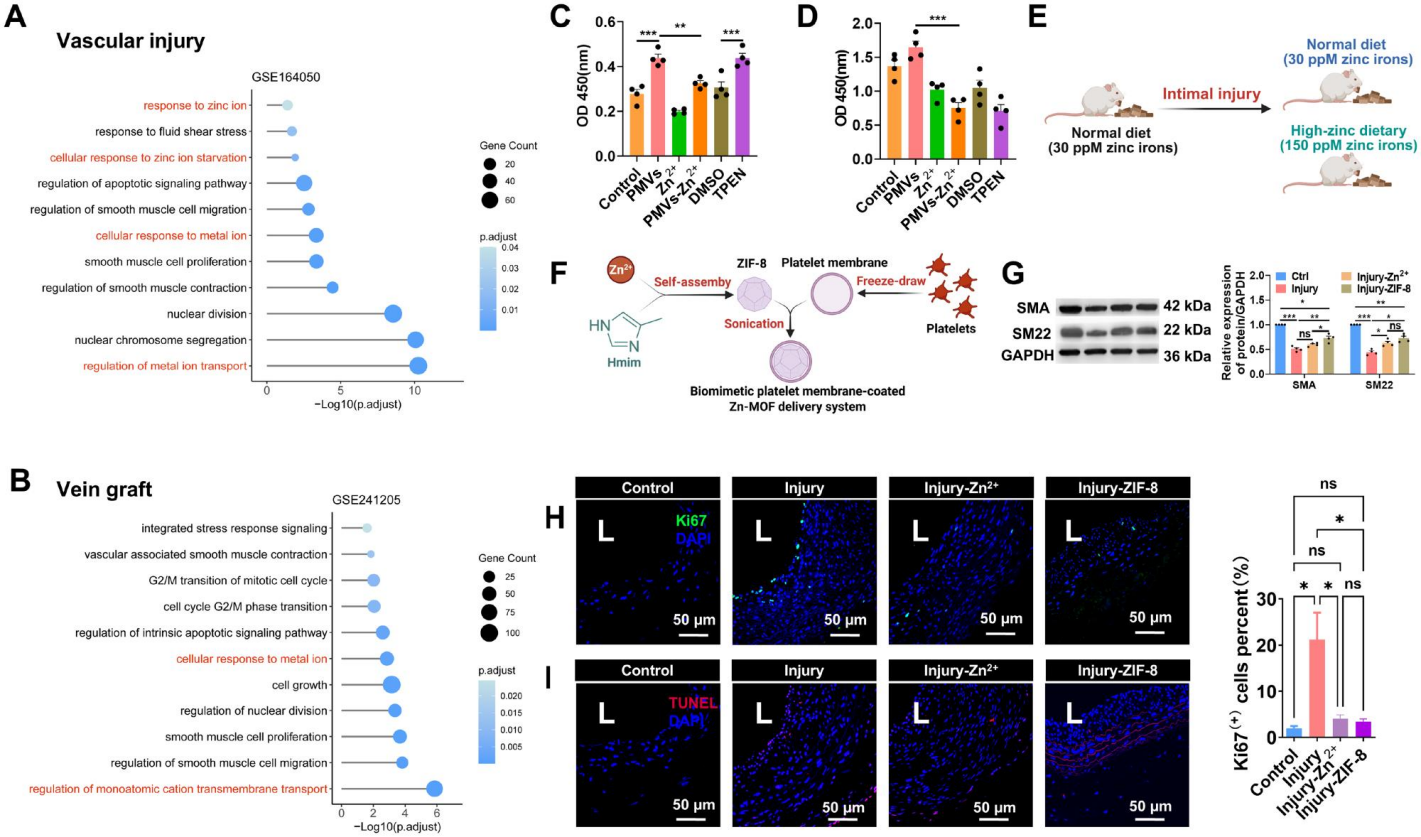
Zinc ion is essential for vascular functions. (**A** and **B**) The analysis of RNA-seq data in rat injured carotid arteries (GSE164050; *n* = 4) and human occluded vein grafts (GSE241205; *n* = 3). Zinc-related pathways were found to be associated with vascular dysfunction (DESeq2; GO enrichment). (**C**) CCK-8 assay evaluated the proliferation rate of VSMCs under different conditions including DMEM only (Control), PMV treatment, zinc supplementation (Zn^2+^), PMVs with zinc supplementation (PMVs-Zn^2+^), DMSO control (DMSO), and TPEN treatment. The data are shown as mean ± SD, ***P < 0.001 (one-way ANOVA with Bonferroni’s multiple comparison *post hoc* test). *n* = 4. (**D**) ELISA assay measured the cleaved caspase-3 levels in VSMCs under respective conditions. The data are shown as mean ± SD, ***P < 0.001 (one-way ANOVA with Bonferroni’s multiple comparison *post hoc* test). *n* = 4. The schematic diagrams depict the two zinc supplementation strategies: (**E**) administration of high-zinc diet and (**F**) fabrication process of platelet membrane-cloaked Zn-MOF nanoparticles (ZIF-8). (**G**) Western blot analyzed the expression of VSMC contractile markers, α-SMA and SM22. The data are shown as mean ± SD, *P < 0.05, **P < 0.01, ***P < 0.001, ns = not significant (one-way ANOVA with Bonferroni’s multiple comparison *post hoc* test). *n* = 4. (**H**) Representative immunofluorescence images of Ki-67 (green) and DAPI (blue) showing that injury-induced cell proliferation was attenuated by high-zinc diet or ZIF-8 supplementation. Quantification of Ki67^+^ cells is presented at right. *n* = 4. (**I**) Representative immunofluorescence images of TUNEL+ (red) and DAPI (blue) indicating that injury-induced cell apoptosis was alleviated by high-zinc diet or ZIF-8 supplementation. *n* = 4. L, lumen.

To investigate the impact of vascular injury on ion homeostasis and cellular responses, we retrieved two gene expression datasets from the GEO database (https://www.ncbi.nlm.nih.gov/geo/): GSE164050 (intimal injury model) and GSE241205 (vein graft dataset). Analysis of these datasets revealed a significant association between zinc ion dysregulation and post-injury vascular dysfunction (Figure 5A). Furthermore, dyshomeostasis of metal ions was implicated in cellular dysfunction within vein grafts (Figure 5B). Therefore, we explored the role of Zn^2+^ both *in vitro* and *in vivo*. The C-caspase 3 ELISA assay shows that PMVs and TPEN (an intracellular membrane-permeable zinc ion chelator) significantly promote SMC apoptosis, while Zn^2+^ supplementation reduces cell apoptosis, suggesting that Zn^2+^ plays a crucial role in inhibiting SMC apoptosis (Figure 5C). CCK-8 assay (Figure 5D) demonstrated that PMVs significantly induce SMC proliferation while Zn^2+^ supplementation reduces cell proliferation, suggesting that Zn^2+^ plays a key role in suppressing SMC proliferation.

To investigate zinc supplementation’s potential in mitigating vascular dysfunctions *in vivo*, we implemented two distinct intervention strategies: a high-zinc dietary regimen (Figure 5E) and a biomimetic platelet membrane-coated Zn-MOF delivery system (ZIF-8, Figure 5F). Western blot analysis (Figure 5G) revealed that the expression of SMC contractile markers, such as alpha-smooth muscle actin (α-SMA) and SM22, is significantly decreased in the injured vascular tissue compared to the control. Notably, Zn^2+^ supplementation enhanced the upregulation of both markers, with the highest expression observed in the Injury*-ZIF-8* group. This suggests that Zn^2+^ released from ZIF-8 nanoparticles actively promotes SMC differentiation in response to vascular injury. In Figure 5H, Ki67 immunofluorescence staining (green) was used to assess cell proliferation. The injury group showed a significant elevation in Ki67-positive cells compared to the control, but both Zn^2+^ supplementation (Injury-Zn^2+^) and ZIF-8 treatment (Injury-ZIF-8) reduced increased Ki67 expression, suggesting that these treatments inhibited SMC proliferation following injury. Besides, the injury group exhibited a higher number of TUNEL-positive cells, whereas Zn^2+^ and ZIF-8 treatments significantly reduced apoptosis, with ZIF-8 showing the most pronounced protective effect (Figure 5I). Collectively, Zn^2+^ regulates SMC proliferation, differentiation and apoptosis both *in vitro* and *in vivo*.

### Fabrication and characterization of ZnO-loaded PCL electrospun external stents (PCL–ZnO stent)

**Figure 6.**
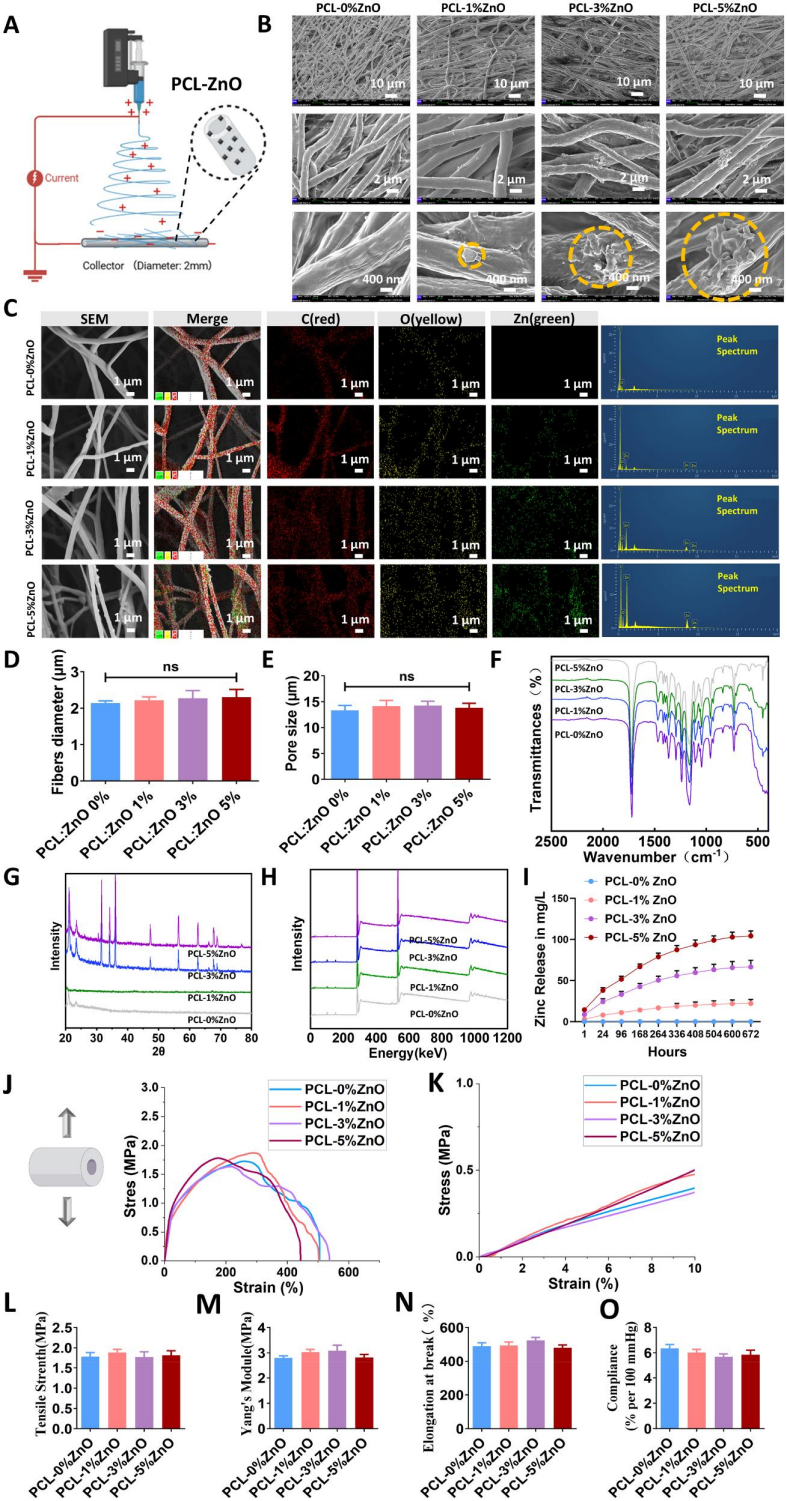
Fabrication and characterization of PCL–ZnO electrospun external stents. (**A**) Schematic diagram of the electrospinning setup used for stent fabrication. (**B**) Representative SEM images show the fiber morphology of stents with 0 wt%, 1 wt%, 3 wt% and 5 wt% ZnO contents at various magnifications. (**C**) Elemental mapping (Zn in green) confirms ZnO distribution in the fibers. (**D** and **E**) Quantitative analysis of fiber diameter (**D**) and pore size (**E**) across different ZnO loadings, revealing no significant differences. *n* = 3. (**F**) FTIR spectra demonstrating characteristic peaks of PCL and ZnO incorporation. (**G**) XRD patterns indicating crystallinity of PCL and ZnO phases. (**H**) XPS analysis confirming the presence of Zn–O bonds. (**I**) Zinc ion release profiles over time, showing controlled, concentration-dependent release behavior. *n* = 3. (**J** and **K**) Stress–strain curves and mechanical properties. (**L**–**N**) quantification of stents’ mechanical parameters *in vitro*. (**O**) Compliance test of the stents under physiological pressure. The data are shown as mean ± SD, with significance indicated as ns = not significant, analyzed by one-way ANOVA with Bonferroni’s *post hoc* test.

PCL, a widely studied semicrystalline polyester known for its remarkable biocompatibility in both *in vitro* and *in vivo* settings [35–42], was selected as the base material due to its hydrolytically degradable ester bonds and Food and Drug Administration-approved clinical applications. In this study, PCL and PCL–ZnO fibrous stents with varying concentrations of ZnO were successfully fabricated using the electrospinning technique (Figure 6A). SEM images showed continuous, bead-free fibers with a random orientation (Figure 6B), forming highly porous networks with interconnected voids. Such porosity is beneficial for applications such as providing optimized permeability for nutrient/waste exchange, controlled fibrotic integration for mechanical support, and guided tissue ingrowth to reinforce the venous wall [43, 44]. Figure 6C presents SEM images and elemental maps of Carbon (red), Oxygen (yellow) and Zinc (green) for PCL fibers containing 0 wt%, 1 wt%, 3 wt% and 5 wt% ZnO. EDS mapping confirms uniform Zn distribution within the fiber matrix at 1 wt% and 3 wt% ZnO, demonstrating effective incorporation during fabrication. At 5 wt% ZnO, minor Zn aggregation is observed, consistent with higher filler loading effects. ImageJ analysis revealed that the average diameter of the PCL nanofibers was 2.23 ± 0.56 μm (Figure 6D), with pore sizes of 13.26 ± 3.33 μm (Figure 6E). Notably, the addition of ZnO at different concentrations did not significantly alter the overall morphology of the stents. After ZnO incorporation, some nanoparticles were observed to be embedded within the fibers, while others formed aggregated clusters on the fiber surfaces, particularly evident in stents with 5 wt% ZnO.

Figure 6F presents the FTIR spectra of PCL and PCL–ZnO fibrous stents. The spectra of PCL exhibited characteristic peaks at 2945 and 2867 cm^−1^, corresponding to the C–H stretching of saturated carbons [45, 46]. A prominent peak at 1724 cm^−1^ indicated the stretching of the carbonyl (–C=O) groups associated with the ester functionality of PCL [47]. The crystalline phase C–C stretching was observed at 1294 cm^−1^ [47], while the asymmetric and symmetric stretching of the C–O–C groups appeared at 1239 and 1168 cm^−1^, respectively [48]. Additionally, peaks within the 500–700 cm^−1^ region corresponded to the stretching vibrations of Zn–O bonds [47]. Figure 6G displays the 2D XRD patterns of PCL and PCL–ZnO fibrous stents. The XRD pattern of pure PCL stents exhibited two distinct diffraction peaks at 2θ values of approximately 21.69° and 24.07°, corresponding to the (110) and (200) planes of the orthorhombic crystal structure, indicating the crystalline nature of the PCL stent [45, 49, 50]. The incorporation of ZnO resulted in the appearance of a peak at approximately 2θ = 56.5°, with the intensity and area of this peak increasing as the ZnO concentration increased. Figure 6H shows the high-resolution X-ray photoelectron spectroscopy spectra of zinc and oxygen in the PCL and PCL–ZnO fibrous stents. The dominant peak centered at 530.2 eV was attributed to the Zn–O bonds in ZnO.

To further evaluate the degradation behavior and zinc release profiles of the PCL–ZnO stents, the stents were incubated in a simulated body fluid environment for 28 days. Zinc ion release was positively correlated with the ZnO loading content (Figure 6I). The stent containing 5 wt% ZnO exhibited a rapid burst release phase (reaching 14.4 mg/L within 0–6 h), followed by sustained release over time (cumulative release of 104.3 mg/L at 672 h). The 3 wt% ZnO stent showed a more moderate release profile (34 mg/L at 72 h, reaching 66.6 mg/L at 672 h). The 1 wt% ZnO stent exhibited the lowest and most stable release (22.2 mg/L at 672 h). The 0 wt% ZnO control group showed no zinc release, confirming the reliability of the system. All stents reached a stable, sustained-release plateau after 72 h of incubation.

Figure 6J–N demonstrated that incorporating varying concentrations of ZnO (0 wt%, 1 wt%, 3 wt%, 5 wt%) into PCL nanofiber stents does not significantly alter their mechanical properties. The stress–strain curves (Figure 6J) and initial elastic region (Figure 6K) show consistent patterns across all groups, indicating comparable elasticity and tensile behavior. Quantitative analysis further confirms that tensile strength (Figure 6L), Young’s modulus (Figure 6M) and elongation at break (Figure 6N) remain statistically unchanged, suggesting that ZnO incorporation preserves the mechanical integrity of the electrospun stents. Compliance test under physiological pressure (Figure 6O) also showed similar values among all groups, indicating that the fundamental mechanical properties required for vascular applications were maintained regardless of ZnO addition.

Collectively, these results demonstrate that we successfully fabricated PCL-based nanofibrous stents with tunable ZnO incorporation, preserving the structural integrity and enhancing the potential bioactivity of the stents.

### PCL–ZnO stent alleviates vascular remodeling and enhances functional arterialization of vein grafts

**Figure 7.**
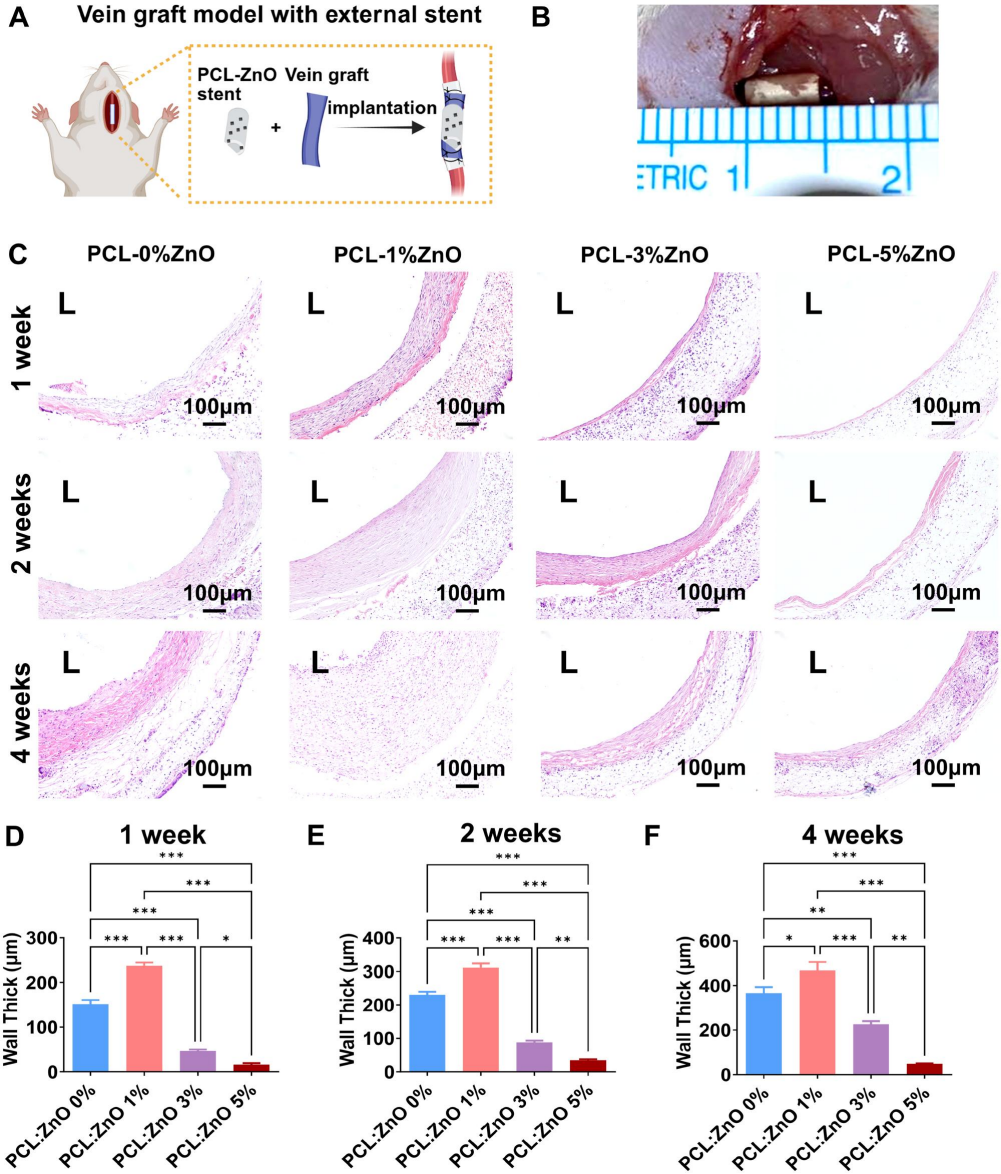
*In vivo* effects of PCL–ZnO stents on rat vein graft remodeling. (**A**) Schematic diagram of external stent placement around a vein graft. (**B**) Intraoperative photograph showing the external stent positioned around the vein graft. (**C**) Representative H&E-stained cross sections of vein grafts from each stent group (PCL-0 wt% ZnO, PCL-1 wt% ZnO, PCL-3 wt% ZnO, PCL-5 wt% ZnO) at 1, 2 and 4 weeks after implantation. (**D**–**F**) Quantitative analysis of vessel wall thickness at 1 week (**D**), 2 weeks (**E**), and 4 weeks (**F**) post-implantation. The data are shown as mean ± SD, with significance indicated as *P < 0.05, **P < 0.01, ***P < 0.001, analyzed by one-way ANOVA with Bonferroni’s *post hoc* test.

To investigate the effects of external stents loaded with different concentrations of zinc ions on vein graft remodeling, we established a vein graft model in which external stents were positioned around the vein grafts to achieve localized sustained release of zinc ions (Figure 7A and B).

Histological assessments were conducted at various time points following implantation to evaluate vascular remodeling. At 1-week post-implantation, H&E staining (Figure 7C) revealed neointimal hyperplasia in the PCL-0% ZnO and PCL-1% ZnO groups. In contrast, the PCL-3 wt% ZnO and PCL-5 wt% ZnO groups showed negligible or no neointimal formation at this early stage. These findings were quantitatively supported by morphological analysis (Figure 7D). At 2-week post-implantation, the PCL-0 wt% ZnO and PCL-1 wt% ZnO groups displayed progression of neointimal hyperplasia, consistent with the typical pathological features of vein graft. In the PCL-3 wt% ZnO group, the vessel wall remained thin, and the structural integrity was preserved. Interestingly, the PCL-5 wt% ZnO group maintained thin vessel walls; however, the near-absence of cellular structures suggested cytotoxic effects due to excessive ZnO release (Figure 7C and E). By 4-week, the PCL-0 wt% ZnO and PCL-1 wt% ZnO groups exhibited exacerbated neointimal thickening. In contrast, the PCL-3 wt% ZnO group showed a well-preserved vascular architecture. Notably, the PCL-5 wt% ZnO group exhibited complete disintegration of vascular architecture and an acellular matrix, strongly suggesting that excessive ZnO concentrations induce cytotoxicity, thereby suppressing cellular proliferation and compromising structural integrity (Figure 7C and F).

Therefore, venous arterialization was better found in PCL-3 wt% ZnO groups after 4-week implantation.

### RNA-Seq analysis of vein grafts treated with PCL-3% ZnO stent

**Figure 8.**
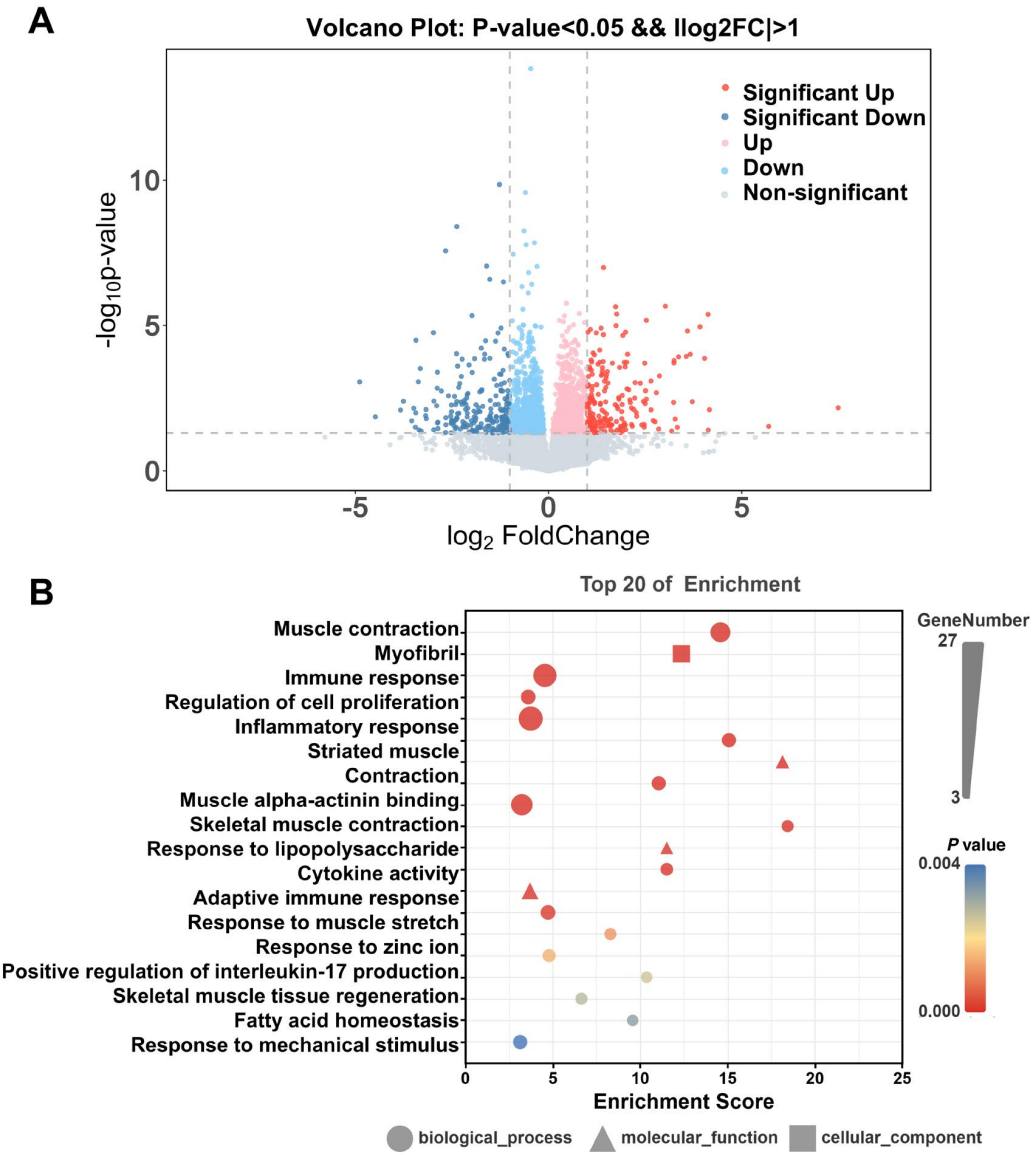
Transcriptomic analysis of vein grafts treated with PCL-3%ZnO external stents at 2 weeks. (**A**) Volcano plot of differentially expressed genes (DEGs), highlighting upregulated (red) and downregulated (green) genes in PCL-3 wt% ZnO group vs. PCL-0 wt% ZnO group (*P* < 0.05). *n* = 3. (**B**) GO enrichment analysis of the top 20 biological processes, revealing important pathways related to muscle contraction, cell proliferation, zinc ion response, etc.

To elucidate the molecular mechanisms underlying vascular remodeling and appropriate arterialization of vein grafts modulated by PCL-3 wt% ZnO external stents, we performed RNA-seq on vein graft samples harvested 2 weeks post-implantation. Volcano plot analysis identified numerous DEGs between the two groups (|log_2_ (FC)| > 1, *P* < 0.05), suggesting that the incorporation of 3 wt% ZnO markedly alters the transcriptional landscape of the vein grafts (Figure 8A). GO enrichment analysis of the top 20 enriched pathways (Figure 8B) demonstrated that the DEGs were significantly associated with biological processes including muscle contraction, regulation of cell proliferation, immune response, inflammatory response and response to zinc ions. The genes involved in muscle contraction and myofibril organization are consistent with the histological observation of well preserved and organized SMC layers in the PCL-3 wt% ZnO group, suggesting a shift toward a functional SMCs phenotype. Conversely, the genes related to inflammatory and immune responses imply a dampening of pro-inflammatory signaling, which likely contributes to the observed suppression of neointimal hyperplasia in the PCL-3 wt% ZnO group. Importantly, we also identified significant enrichment of metabolic pathways, including fatty acid homeostasis and antioxidant activity. This suggests that zinc incorporation modulates metabolic processes within the vein graft, potentially shifting cellular energy metabolism and redox balance. Such metabolic reprogramming could be critical for reducing pathological neointimal proliferation and supporting a more quiescent vascular phenotype. Furthermore, the enrichment of pathways associated with cytokine activity and adaptive immune responses indicates a dampening of excessive inflammation, likely contributing to the attenuation of neointimal hyperplasia.

Together, these transcriptomic findings highlight that 3 wt% ZnO external stents exert a multifaceted influence on vein graft biology, not only suppressing pro-inflammatory signaling and pathological proliferation but also maintaining metabolic homeostasis.

### PCL-3% ZnO stent modulates SMC spatial organization and phenotype

**Figure 9.**
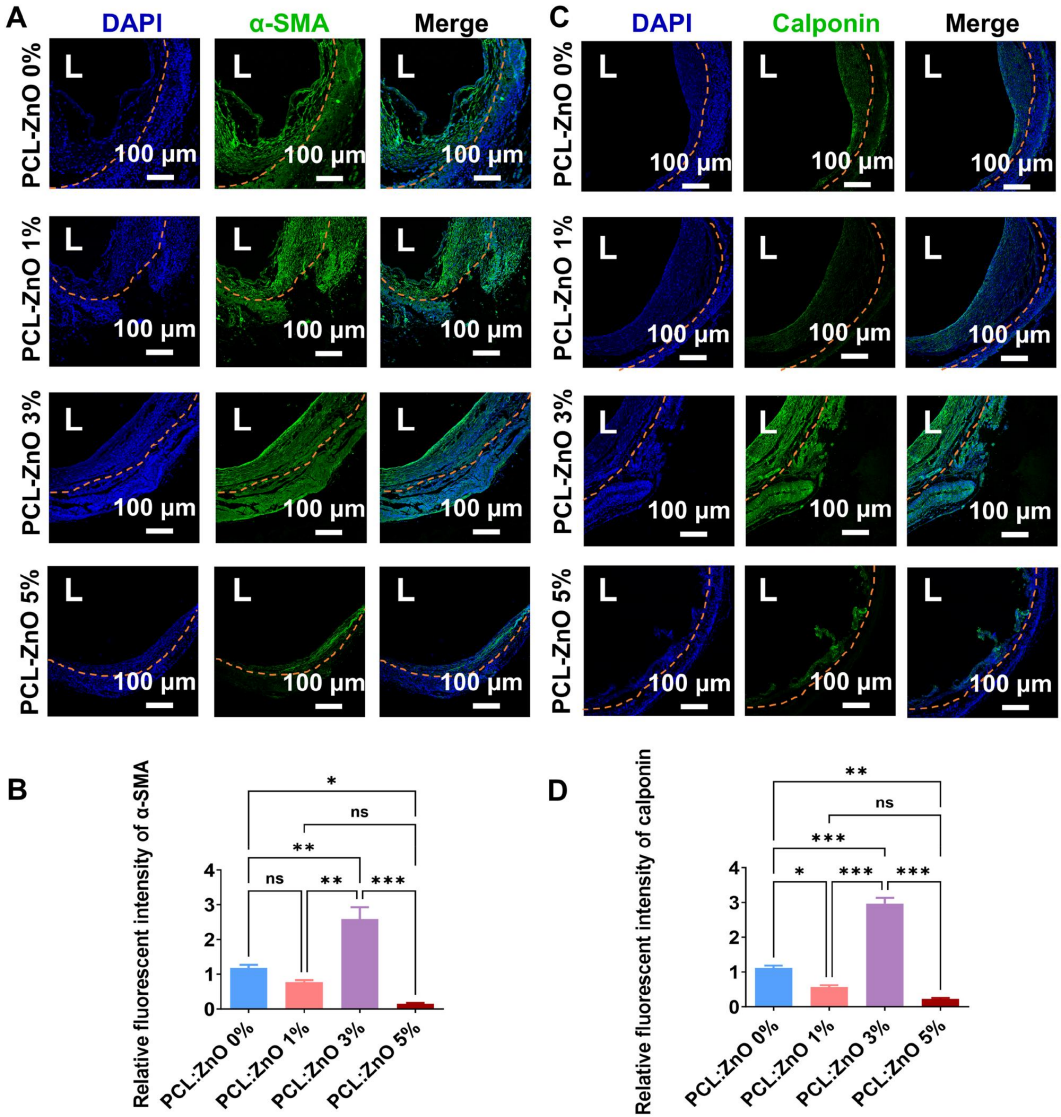
Immunofluorescence analysis of SMC distribution and phenotype in 4 W vein grafts implanted with external stents containing different ZnO concentrations. (**A**) Representative immunofluorescence images of vein graft cross sections stained for α-SMA (green), with nuclei counterstained with DAPI (blue). (**B**) Quantification of α-SMA fluorescence intensity. (**C**) Representative images of Calponin (green) staining in vein graft cross sections, with DAPI (blue) nuclear counterstaining. (**D**) Quantification of Calponin fluorescence intensity. The data are shown as mean ± SD, ns, not significant, with significance indicated as *P < 0.05, **P < 0.01, ***P < 0.001, analyzed by one-way ANOVA with Bonferroni’s *post hoc* test.

To further validate the roles of PCL–ZnO stents in venous arterialization after vein grafting, immunofluorescence staining of α-SMA and Calponin was performed on sections harvested from 4-week grafted veins (Figure 9). In PCL-0 wt% ZnO and PCL-1 wt% ZnO groups, α-SMA staining confirmed the presence of SMCs within the neointima and medial layers. However, Calponin expression was relatively low in these groups, indicating a synthetic phenotype of SMCs. The PCL-3 wt% ZnO group showed the strongest expression of α-SMA and Calponin, indicating the presence of greater amounts of contractile SMCs compared to the other groups. Intriguingly, the PCL-3 wt% ZnO group also showed increased α-SMA-positive cell infiltration into the stent (Figure 9A and B), suggesting active cellular recruitment and integration into the stent environment. The SMC infiltration may further contribute to the stabilization of the neointimal structure and mechanical reinforcement of the graft. Similarly, Calponin expression was significantly elevated in the PCL-3 wt% ZnO group, indicating the maintenance of a contractile SMC phenotype (Figure 9C and D). In contrast, the PCL-5 wt% ZnO stent showed significantly diminished α-SMA and Calponin expression (Figure 9A–D), suggesting that excessive ZnO impairs SMC viability and may destabilize vascular wall integrity.

Together, optimal ZnO incorporation (3 wt%) sustains SMC contractility (α-SMA^+^/Calponin^+^). PCL-3 wt% ZnO stent promotes functional arterialization of vein grafts.

### *In vitro* and *in vivo* effects of zinc ions on SMC proliferation and function

**Figure 10.**
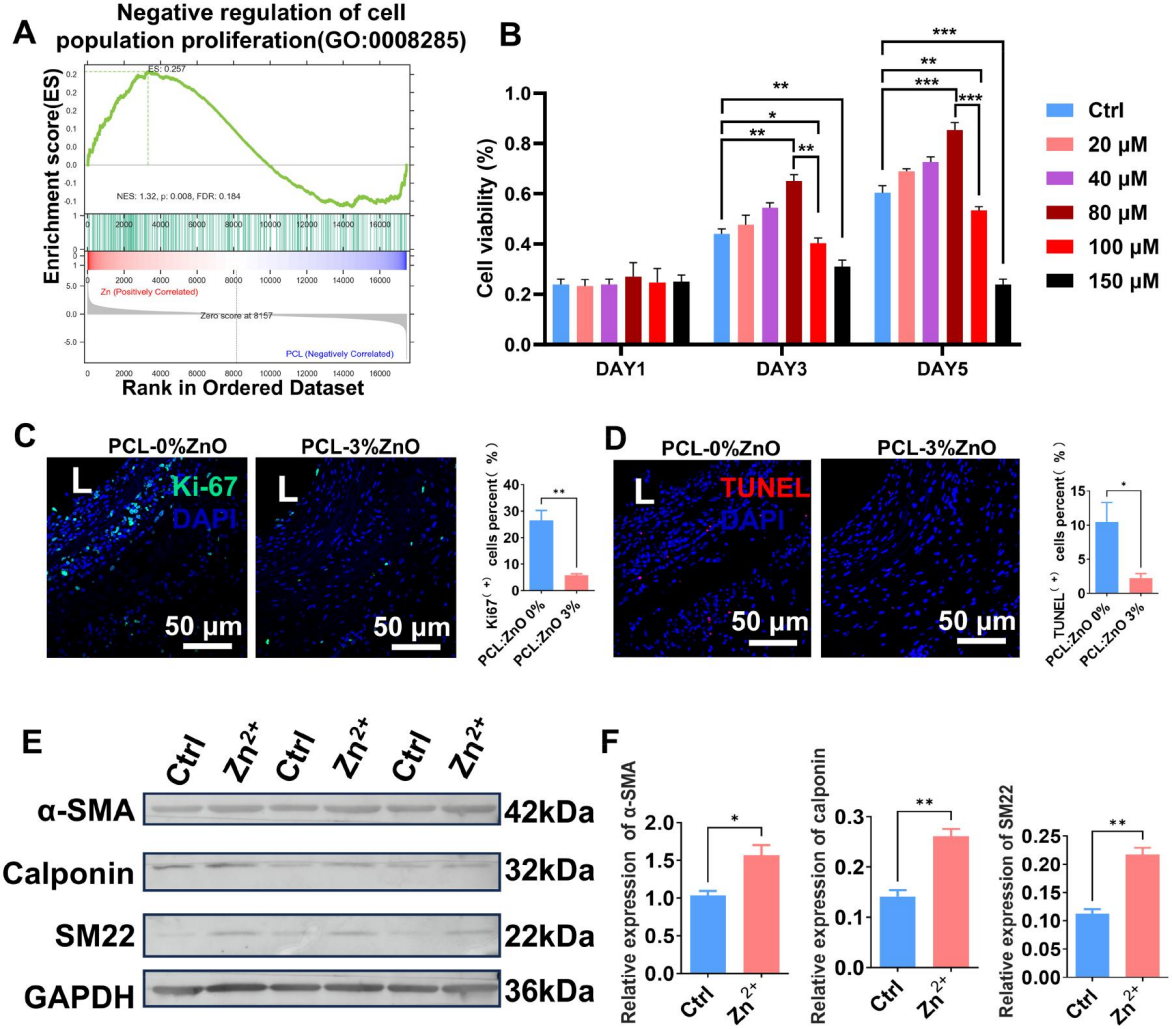
Zinc ions modulate SMC proliferation and apoptosis *in vitro* and *in vivo*. (**A**) GSEA of transcriptomic data from vein grafts at 2 weeks, indicating significant enrichment of pathways related to negative regulation of cell population proliferation. (**B**) *In vitro* cell viability of SMCs exposed to varying Zn^2+^ concentrations (20, 40, 80, 100 and 150 μM) measured by CCK-8 assay at days 1, 3 and 5. *n* = 3. (**C**) Immunofluorescence staining of Ki67 (green) and DAPI (blue) in vein grafts after 4 weeks, revealing a marked reduction in proliferative activity in the PCL-3 wt% ZnO group compared to PCL-0 wt% ZnO group. Quantification of Ki67^+^ cells is presented at right. (**D**) *In vivo* TUNEL (red) staining showed decreased apoptosis levels in PCL-3 wt% ZnO group compared to PCL-0 wt% ZnO group, indicating that 3% Zn^2+^ modulates vascular remodeling primarily through proliferation suppression without inducing apoptosis in vein grafts. Quantification of TUNEL^+^ cells is presented at right. (**E** and **F**) Western blot analysis of SMCs treated with 100 μM Zn^2+^ showing upregulated expression of α-SMA (42 kDa), Calponin (32 kDa) and SM22 (22 kDa). *n* = 3. The data are shown as mean ± SD. Comparisons between two groups were performed using paired Student’s *t*-test, and comparisons among multiple groups were conducted using one-way ANOVA with Bonferroni’s multiple comparison *post hoc* test. Significance was indicated as *P < 0.05, **P < 0.01, ***P < 0.001.

GSEA of RNA-seq data from vein grafts treated with PCL-3 wt% ZnO external stents revealed significant enrichment of “negative regulation of cell population proliferation” (GO: 0008285) (Figure 10A), indicating a suppression of proliferative pathways within the vascular wall microenvironment. *In vitro* cell viability assays demonstrated a biphasic, concentration-dependent response of SMCs to Zn^2+^ treatment (Figure 10B). At moderate concentrations (20–80 μM), Zn^2+^ treatment enhanced cell viability by day 3, with the 80 μM group showing the highest proliferative activity. In contrast, higher concentrations of Zn^2+^ (100 and 150 μM) significantly reduced cell viability on days 3 and 5, indicating a robust anti-proliferative effect at these concentrations. *In vivo* histological analyses further supported these findings. Immunofluorescence staining for Ki67 demonstrated a marked reduction in proliferative activity within the vascular wall in the PCL-3 wt% ZnO group compared to PCL-0 wt% ZnO (Figure 10C). TUNEL staining revealed decreased apoptosis levels in PCL-3 wt% ZnO group compared to PCL-0 wt% ZnO group (Figure 10D). Therefore, Zn^2+^ primarily modulates vascular remodeling through suppression of proliferation rather than induction of cell death. To investigate zinc-induced phenotype switching of SMCs, western blot analysis was performed in SMCs treated with 100 μM Zn^2+^ (Figure 10E). The results showed a significantly elevated expression of α-SMA, SM22 and Calponin in Zn^2+^-treated cells compared to controls (Figure 10F). These data suggest that 100 μM Zn^2+^ enhances SMC differentiation and suppresses SMC proliferation.

Collectively, these results establish a concentration-dependent role of zinc ions in SMC behavior. Moderate ZnO loading (3 wt%) effectively mitigated neointimal hyperplasia and promoted appropriate venous arterialization via modulating SMC proliferation and phenotype switching.

### Metabolomic analysis of vein grafts treated with PCL-3 wt% ZnO stent

**Figure 11.**
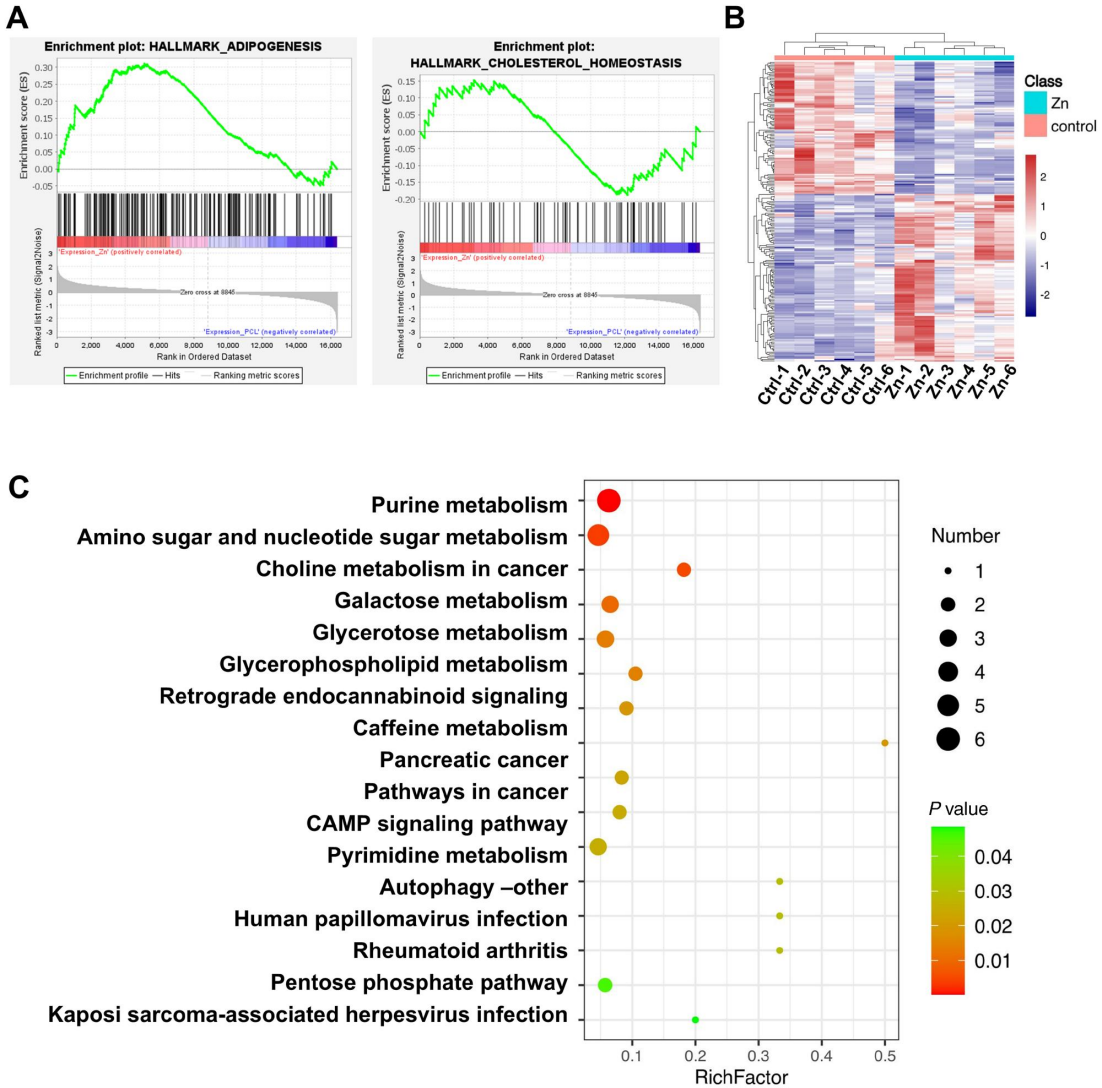
Metabolomic profiling of vein grafts implanted with PCL-3 wt% ZnO external stents. (**A**) GSEA of RNA-seq data from PCL-3 wt% ZnO external stents treated vein grafts at 2 weeks, showing significant enrichment of gene sets involved in lipid biosynthesis (HALLMARK_ADIPOGENESIS) and cholesterol homeostasis (HALLMARK_CHOLESTEROL_HOMEOSTASIS). (**B**) Hierarchical clustering heatmap of metabolomic profiles from 4-week vein grafts, revealing distinct metabolite expression patterns in the PCL-3 wt% ZnO group compared to controls. (**C**) Pathway enrichment analysis of differential metabolites, identifying key metabolic pathways including purine metabolism, amino sugar and nucleotide sugar metabolism, galactose metabolism, and glycerophospholipid metabolism.

Since PCL-3 wt% ZnO stent promotes functional arterialization of vein grafts, we performed the metabolomic profiling of vein grafts implanted with PCL-3 wt% ZnO external stents. Altered lipid metabolic pathways were found in the PCL-3 wt% ZnO group (Figure 11A). Comprehensive untargeted metabolomic profiling was conducted to elucidate the downstream metabolic reprogramming in vein grafts 4 weeks post-implantation. Hierarchical clustering analysis (Figure 11B) revealed distinct metabolic signatures in the PCL-3 wt% ZnO stent group compared to controls, reflecting substantial alterations in the biochemical landscape of the vascular wall.

GO analysis (Figure 11C) demonstrated significant alterations in key metabolic pathways, including purine metabolism, amino sugar and nucleotide sugar metabolism, choline metabolism and glycerophospholipid metabolism. These pathways are intimately linked to nucleotide turnover, membrane phospholipid remodeling and cellular stress adaptation—all critical processes in the context of vascular remodeling and vein graft adaptation to the arterial circulation [51, 52]. Importantly, the observed metabolic reprogramming aligns with the well-established biological roles of zinc ions in cellular homeostasis. Zinc acts as a cofactor for numerous metalloenzymes and transcription factors to modulate enzymatic activity, redox balance and membrane stability [25]. It also directly influences cellular signaling cascades that govern inflammation, oxidative stress responses and extracellular matrix remodeling [30]. In the context of vein graft disease, characterized by neointimal hyperplasia and vessel wall thickening, controlled Zn^2+^ release from PCL-3 wt% ZnO stents may support a metabolic shift toward enhanced membrane integrity (via glycerophospholipid metabolism), reduced oxidative stress (via nucleotide and choline metabolism) and improved cellular survival and phenotype stability.

These metabolomic findings, together with the previous histological evidence of reduced neointimal hyperplasia and enhanced SMCs contractile phenotype, suggest that moderate ZnO incorporation (3 wt%) promotes a coordinated metabolic and phenotypic remodeling of the vascular wall to keep appropriate arterialization of vein grafts.

## Discussion

This study reveals that ZnO incorporation within electrospun PCL external stents exhibits a concentration-dependent, bidirectional regulatory influence on vascular remodeling in a rat vein graft model. Specifically, moderate ZnO loading (3 wt%) effectively mitigated neointimal hyperplasia and promoted appropriate venous arterialization. External stents containing 3 wt% ZnO demonstrated a pleiotropic effect on vein graft biology, including significant suppression of pro-inflammatory pathways, inhibition of maladaptive cellular proliferation and regulation of metabolic homeostasis. These findings highlight the potential of zinc-based external stents to modulate the local biological environment of vein grafts and improve graft patency.

Physiological mechanical stress is essential for proper cardiovascular development and tissue homeostasis. However, aberrant mechanical stimuli, such as excessive cyclic stretch or disturbed flow, act as pathogenic drivers of cardiovascular dysfunction and disease [4, 9, 11, 53, 54]. Both our work and previous studies have demonstrated that abnormal mechanical stress plays a pivotal role in vascular remodeling [4, 9, 17]. For instance, hypertension-induced cyclic overstretch exacerbates vascular remodeling and dysfunction [12, 17, 55], while elevated stretch also contributes to venous remodeling and vein graft failure [4, 6]. Notably, we previously demonstrated that external stents can mitigate pathological vein graft remodeling by providing mechanical support [56]. Building on this foundation, the present study further investigates the synergistic effects of mechanical stress and surgical injury on vascular remodeling. Our findings reveal that moderate ZnO incorporation (3 wt%) in external stents optimally modulates local biological responses by suppressing pathological cell proliferation without triggering apoptosis, thereby facilitating favorable venous arterialization.

Zinc plays essential and concentration-dependent roles in cell biology. Physiological levels of zinc promote cell proliferation, migration and adhesion through its function as a cofactor in numerous metalloenzymes and transcription factors [24, 26]. However, excessive zinc concentrations can induce oxidative stress, mitochondrial dysfunction, platelet function and apoptosis [29, 30, 57]. And oxidative stress, mitochondrial dysfunction and PMVs are all important factors in regulating abnormal VSMCs proliferation, endothelial dysfunctions and vascular remodeling [10, 11, 14, 15, 31, 58–64]. Our *in vitro* experiments further support that low concentrations of Zn^2+^ enhance SMC proliferation, whereas moderate concentrations exert an inhibitory effect. Consistent with our *in vitro* findings, *in vivo* histological assessment and immunofluorescence staining further confirmed that Zn^2+^ exerts concentration-dependent regulatory effects on SMC proliferation. The PCL-3 wt% ZnO composite demonstrated significantly enhanced expression of α-SMA and Calponin markers, suggesting not only increased SMC density but also maintained contractile functionality—a critical factor for both vascular tone regulation and structural stability. Moreover, the presence of α-SMA-positive cells infiltrating the stent structure indicates active cellular recruitment and integration, a process that likely contributes to improved mechanical reinforcement and long-term graft stability [36, 65]. In contrast, elevated ZnO concentrations (5 wt%) markedly reduced cell viability and disrupted tissue architecture, highlighting the critical need for precise modulation of zinc ion levels within the stent microenvironment.

Existing research on endovascular zinc alloys underscores a critical trade-off: while zinc ions exhibit essential bioactivity, their cytotoxic potential necessitates stringent control over release kinetics and local concentrations [66]. Our study bridges this knowledge gap to perivascular applications, showing that engineered Zn^2+^ release from external stents achieves threefold therapeutic modulation: (i) inhibition of pathological hyperplasia, (ii) preservation of vessel wall integrity and (iii) stabilization of SMC contractile phenotype. Collectively, these observations reveal a dose-dependent duality of zinc ions—effective therapeutic modulation within a narrow concentration range, yet deleterious outside it—highlighting the criticality of optimized Zn^2+^ dosing (around 3 wt% loading) across stent platforms and delivery modes. These findings provide critical translational guidance for optimizing zinc-based vascular devices, whether deployed as internal stents or external scaffoldings.

However, this study has several limitations that should be acknowledged. First, the long-term *in vivo* behavior of ZnO-coated stents—including degradation kinetics, zinc ion release dynamics and systemic zinc accumulation—remains uncharacterized, a critical gap for clinical translation. Second, although we observed distinct histological and phenotypic responses to varying ZnO concentrations, the underlying molecular mechanisms remain unclear, necessitating further mechanistic investigation. Lastly, while our findings provide proof-of-concept data in a small-animal model, further validation through large-animal studies and clinical trials will be essential to assess translational potential and safety in human applications.

## Conclusion

Our study demonstrates that an external stent incorporating an optimized concentration (3 wt%) of ZnO effectively mitigates pathological vascular remodeling in vein grafts. This carefully calibrated zinc ion release strategy achieves a dual therapeutic effect: it not only suppresses maladaptive neointimal hyperplasia but also promotes physiological venous arterialization and maintains the contractile phenotype of vascular SMCs. Together, these findings highlight the translational potential of precision-controlled zinc ion modulation as a robust approach to enhancing the long-term patency and functional integrity of vein grafts.
